# Dissociative Electron Attachment to 8‑(Trifluoromethyl)thioadenine: A Potential Radiosensitizer

**DOI:** 10.1021/acs.jpcb.6c03946

**Published:** 2026-07-20

**Authors:** Adrian Szczyrba, Magdalena Datta, Samanta Makurat, Jiakuan Chen, Karol Biernacki, Sebastian Demkowicz, Stephan Denifl, Janusz Rak

**Affiliations:** † Laboratory of Biological Sensitizers, Department of Physical Chemistry, Faculty of Chemistry, 49646 University of Gdańsk, Wita Stwosza 63, Gdańsk 80-308, Poland; ‡ Department of Inorganic Chemistry, Faculty of Chemistry, Gdańsk University of Technology, Narutowicza 11/12, Gdańsk 80-233, Poland; § Institut für Ionenphysik und Angewandte Physik, 27255 Universität Innsbruck, Technikerstraße 25, Innsbruck A-6020, Austria; ∥ Center for Molecular Biosciences Innsbruck, Universität Innsbruck, Technikerstraße 25, Innsbruck A-6020, Austria; ⊥ Department of Organic Chemistry, Faculty of Chemistry, 49557 Gdańsk University of Technology, Narutowicza 11/12, Gdańsk 80-233, Poland

## Abstract

The development of new biological radiosensitizers has primarily focused on halogenated uracil-based derivatives such as 5-bromo-2’-deoxyuridine, and more recently, 5-iodo-4-thio-2′-deoxyuridine or 5-thiocyanato-2′-deoxyuridine. However, further modifications of the uracil/uridine ring are limited due to challenges related to synthesis and stability. In this study, we investigate dissociative electron attachment (DEA) to 8-(trifluoromethyl)­thioadenine (ASCF_3_), proposing a new potential purine-based radiosensitizer for cancer cells. This is explored using a crossed electron-molecule beam (CEMB) experiment and stationary radiolysis in an aqueous environment. To support our findings, we combined experimental results with theoretical calculations employing DFT methods to analyze the energy threshold and DEA profiles. The most abundant anion observed in the CEMB experiments corresponds to [ASCF_3_–CF_3_]^−^, whereas the radiolytic study reveals that the novel derivative undergoes a single decomposition channel in aqueous solution, yielding a cyclic [ASCF_3_–HF] species. These findings highlight that disregarding the influence of water in cellular processes may lead to erroneous mechanistic conclusions.

## Graphical abstract

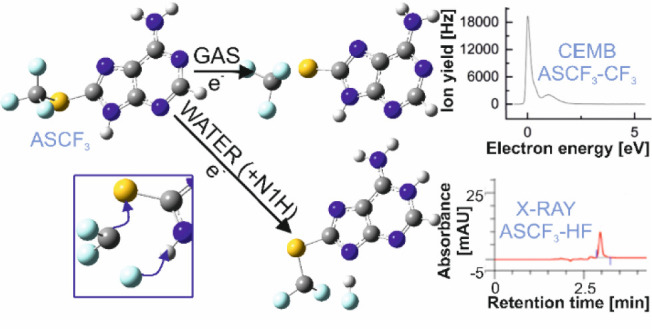

## 1 Introduction

Ionizing radiation (IR) is a widely used modality in cancer treatment.^1^ Its therapeutic efficacy relies on the interaction between cellular DNA and reactive species generated by water radiolysis, predominantly hydroxyl radicals. However, the hypoxic microenvironment, characteristic of solid tumors—accounting for approximately 80% of all cancer cases—significantly reduces IR effectiveness. This phenomenon, known as the “oxygen effect”, renders hypoxic cells about three times less sensitive to radiation compared to normoxic ones.^2^ Furthermore, although IR targets tumor cells, it also poses a risk to the surrounding healthy tissues by inducing mutations that could lead to secondary cancers. To improve the efficacy of IR and selectively sensitize tumor cells, researchers have developed radiosensitizers—compounds that increase DNA susceptibility to radiation-induced damage, especially under hypoxic conditions.^3^

One promising class of radiosensitizers are modified nucleosides (NBs), which exploit the second most abundant product of water radiolysis: low-energy electrons (LEEs).^4^ When incorporated into DNA, these modified nucleosides undergo dissociative electron attachment (DEA) upon interaction with LEEs, leading to products that facilitate DNA strand breaks. A well-known example is 5-bromo-2′-deoxyuridine (BrdU) or 5-iodo-4-thio-2’-deoxyuridine (ISdU) which, upon electron attachment, generates a bromide anion and a reactive uracil-5-yl radical that induces DNA cleavage.^5,6^ Most existing radiosensitizing nucleosides are uridine derivatives,^7^ while purine analogs remain underexplored. Given the potential of purines to expand the limited pool of effective radiosensitizers, recent studies have focused on synthesizing and characterizing novel purine-based derivatives.^8^

Adenine, a purine nucleobase, is less chemically reactive than uracil, but substituting its C8 position has shown promise in facilitating Dissociative Electron Attachment (DEA). Notably, 8-bromoadenine^9–12^ and 8-bromoguanine^13,14^ exhibit strong susceptibility to damage induced by electron attachment.^15,16^ To develop alternative purine-based radiosensitizers, we synthesized 8-(trifluoromethyl)­thioadenine (ASCF_3_), hypothesizing that the electron-withdrawing effects of the trifluoromethyl group would promote efficient DEA.^17^ Gas-phase experiments using crossed electron-molecule beam (CEMB) techniques, combined with quantum-chemical (QM) calculations, confirm the high propensity of ASCF_3_ for electron attachment and subsequent fragmentation. Moreover, aqueous-phase studies demonstrate the expected electron-attachment-induced damage. Hence, the current paper reports on the synthesis and the electron-attachment-induced dissociation of ASCF_3_ in both gaseous and aqueous phases, emphasizing the role of chemical modifications in enhancing DNA susceptibility to electron-attachment-driven damage.

## 2 Methods

### 2.1 Reagents

All listed reagents were commercially available and used as delivered without any further purification: adenine (Ambeed), bromine (Avantor Performance Materials Poland, formerly known as Polish Chemical Materials, POCH), potassium hydroxide (POCH), thiourea (Sigma-Aldrich, Merck), trifluoroiodomethane (Thermo Scientific (Alfa Aesar)). Water that was used was distilled water. Used solvents were bought from POCH with reagent-grade purity, unless otherwise noted: acetic acid, acetone, concentrated ammonia solution, butan-1-ol, dichloromethane (DCM), diethyl ether, methanol (MeOH), *N*,*N*-dimethylformamide (DMF). Anhydrous solvents were obtained by standard procedures described in the literature and were stored under 4Å molecular sieves. Laboratory glassware was cleaned and dried before use. Anhydrous conditions were acquired by drying laboratory glassware with a Bunsen burner and cooling under the flow of a technical argon. Column chromatography was performed using irregular silica gel (230–400 mesh, 60Å, SiliCycle).

### 2.2 Analytical Methods

^1^H NMR spectra were acquired on a Varian Inova 500 (500 MHz) spectrometer at a sample temperature of 25 °C and were visualized using MestReNova v.14.0.0 (Mestrelab Research). NMR chemical shifts are reported in ppm and are referenced to DMSO-d_6_ at 2.50 ppm and are noted as such. Multiplets are described as follows: s – singlet, bs – broad singlet.

MS and MS/MS analyses were carried out on a TripleTOF 5600+ high resolution hybrid quadrupole time-of-flight mass spectrometer (SCIEX, Framingham, MA, USA) equipped with an electrospray ionization (ESI) source (DuoSpray) and coupled to a Nexera X2 ultrahigh-performance liquid chromatography (UHPLC) system. The instrument was operated in negative ion mode with an ion spray voltage of −4.5 kV, curtain gas set to 25 psi, ion source gas 1 and gas 2 set to 25 and 15 psi, respectively, and a source temperature of 300 °C. Data were acquired in information-dependent acquisition (IDA) mode over an *m*/*z* range of 100–1000 for TOF MS and 50–1000 for product ion spectra, using a TOF MS accumulation time of 100 ms and selecting the most intense precursor ions for MS/MS acquisition. Precursor ions were isolated with an isolation window of 0.5 Da and subjected to collision induced dissociation in the collision cell using a collision energy (CE) of 10 eV to generate high resolution MS/MS spectra. Raw data were processed using Analyst software (version 1.7.1, SCIEX) for instrument control and data acquisition and PeakView (version 2.1, SCIEX) for peak detection, accurate mass extraction, and assignment of fragment ions based on theoretical isotope patterns and MS/MS fragmentation rule.

Chromatographic separation was carried out on a Kinetex C18 column (Phenomenex, 2.1 × 150 mm, 2.6 μm, 100 Å). The mobile phase consisted of solvent A (0.1% HCOOH in deionized water) and solvent B (80% ACN). Elution was performed using a linear gradient from 0 to 30% of phase B over 20 min at 25 °C, with a flow rate of 0.3 mL·min^–1^ and an injection volume of 10 μL. The eluent was diverted to waste during the first minute after injection.

### 2.3 Synthesis

The initial two steps of ASCF_3_ synthesis (see Scheme 1) have been previously described by Borrmann^18^ and Janeba.^19^ 8-bromoadenine (1) was obtained with 98% yield (for ^1^H NMR see Figure S1 in Supporting Information) while 6-amino-7,9-dihydro-8H-purine-8-thione (2) with 48% yield (for ^1^H NMR see Figure S2). In the final step, leading to 8-(trifluoromethyl)­thioadenine, a 100 mL Bunsen burner-dried round-bottom flask, equipped with an glass stopcock vacuum adapter, was charged with 40 mL of anhydrous DMF, 2 (1 eq, 0.00359 mol, 0.600 g) and KOH (2 eq, 0.00718 mol, 0.369 g). Subsequently, CF_3_I (4 eq, 0.01440 mol, 2.813 g) was introduced via a preweighted ballon through the adapter. The stopcock was opened and the mixture was stirred at room temperature overnight. After this period, the gas dissolved in a solvent, and the mixture was stirred in a tightly closed system at 90 °C for 72 h. Next, the solvent was evaporated under reduced pressure, and the residue was subjected to preliminary purification by column chromatography using a DCM:MeOH gradient elution system (1:0 → 20:1). Obtained yellowish solid, was then purified with semipreparative HPLC (Shimadzu, LC 20AD) and analytes were separated on a Synergy Polar-RP (Phenomenex) reverse-phase column (10 × 250 mm, 4 μm in particle size and 100 Å in pore size) at a flow rate 4 mL·min^–1^. The program was set to linear gradient 0–80% of phase B in 20 min (mobile phase A: 0.1% HCOOH in water, B: 80% ACN. The compound’s structure was confirmed via ^1^H NMR spectroscopy and mass spectrometry (see Figures S3–S5). At the same time, the purity of the obtained product (98%) was verified by HPLC analysis (see Figure S6). The product was received as a white solid (m = 0.236 g, n = 0.001 mol) with 28% yield.

**Scheme 1:**
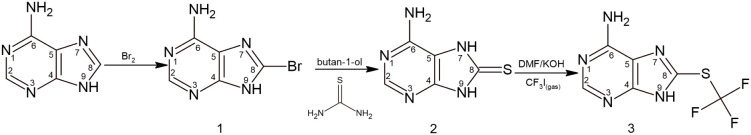
8-(Trifluoromethyl)­Thioadenine Synthesis

### 2.4 Crossed Electron-Molecular Beam Experiment

A comprehensive description of the experimental setup used to investigate dissociative electron attachment (DEA) to ASCF_3_ in the gas phase was provided in previous article by Denifl and coworkers.^6,12,20,21^ The apparatus primarily consisted of a crossed electron–molecular beam system coupled with a quadrupole mass spectrometer. The ASCF_3_ vapor was produced by heating solid ASCF_3_ in a resistively heated copper oven located within a vacuum chamber. The experiment was conducted under high-vacuum conditions, maintaining a chamber pressure of 8 × 10^–8^ mbar and a sample temperature of 356 K. The sample vapor was introduced into the interaction region of a hemispherical electron monochromator (HEM) through a 1 mm diameter capillary, where it intersected perpendicularly with a monochromatized electron beam. The HEM was operated with an energy resolution of approximately 130 meV (full width at half-maximum, FWHM) and an electron current of approximately 30 nA. Anions formed upon electron attachment to ASCF_3_ were extracted from the interaction zone by a weak electrostatic field and directed into a quadrupole mass filter, where they were separated based on their mass and subsequently detected using a channeltron electron multiplier operating in single-pulse counting mode. The ion yield of each anion was recorded as a function of the incident electron energy. Prior to measuring the negative ion yield, an electron impact ionization mass spectrum was obtained at 70 eV across different temperatures to confirm the absence of significant thermal decomposition at the selected sublimation temperature. The electron energy scale and resolution were calibrated using the well-characterized Cl^–^ yield from CCl_4_ at 0 eV.^22^ Electrons that traversed the interaction region without undergoing attachment were collected on a Faraday plate and monitored using a picoamperometer.

### 2.5 Stationary Radiolysis in an Aqueous Solution

The radiolysis of a 10^–4^ M solution of 8-(trifluoromethyl)­thioadenine was conducted in Eppendorf tubes with the addition of 0.03 M tert-butanol as a scavenger for hydroxyl radicals (^•^OH) and a phosphorus buffer (10 mM, pH 7). The irradiation was performed using a CellRad X-ray Cabinet (Faxitron X-ray Corporation) at an X-ray tube voltage of 130 kV and a current of 0.1 mA. A 0.5 mm aluminum filter was applied. Prior to irradiation, the samples were deoxygenated by argon purging for 3 min. To ensure the formation of sufficient concentrations of radiolysis products for reliable analytical detection and characterization, the exposure dose was 500 Gy, delivered at a rate of 5.83 Gy/min. This dose, typically used in experimental settings, enables mechanistic investigation of radiation-induced degradation pathways. After irradiation, samples were analyzed with HPLC analysis with using of reversed-phase RP-HPLC high-performance liquid chromatography, using a Dionex Ultimate 3000 chromatograph (Thermo Scientific) and a Wakopak Handy ODS analytical column (150 × 4.6 mm). The mobile phase was a system of solvents A and B, applied in a 20 min gradient of 0–30% phase B, where A – 0.1% HCOOH in water; B – 80% ACN. The volumetric flow rate of eluents was 1 mL·min^–1^. Detection was carried out at a wavelength of 260 nm.

### 2.6 Computational Methods

#### 2.6.1 Gas-Phase Calculations

To predict the CEMB thresholds, the geometries of all species involved in the studied fragmentations were fully optimized at the M06-2X^23^ level using the aug-cc-pVTZ^24,25^ basis set. Harmonic frequencies were calculated by diagonalizing the Hessian matrix, which contains the second derivatives of the total energy with respect to nuclear coordinates. All optimized geometries were verified to be geometrically stable minima (all force constants positive). The Gibbs Free Energies were used to determine vertical attachment energies (VAE) and adiabatic electron affinities (AEA). The electronic energies (E) corrected for zero point energy (ZPE) were employed in calculations of thermodynamic thresholds.

#### 2.6.2 Water-Phase Calculations

These calculations were conducted at the M06-2X^23^/6-31++G­(d,p)^26,27^ level using the Polarizable Continuum Model (PCM)^28^ of water. All the optimized geometries were geometrically stable: harmonic frequency analysis confirmed positive force constants for the minima and a single negative force constant for the first-order transition state. The intrinsic reaction coordinate (IRC) procedure^29^ was employed to ensure that the identified transition state connects the correct minima.

The *pK_a_* of ASCF_3_ was calculated according to formula:^30^

$$pK_{a}=pK_{ref}-\frac{\Delta \Delta G}{2.303RT}$$

Where *pK_ref_* is the negative standard logarithm of the *K_a_* constant for the reference compound, ASCH_3_,^8^ *R* is the gas constant, *T* is the temperature, and ΔΔ*G* represents the difference in free energies of the base and its conjugate acid for the studied compound/reference compound pair.

$$ASCF_{3}^{-}+ASCH_{3}\overset{\Delta \Delta G}{↔}ASCF_{3}+ASCH_{3}^{-}$$

All quantum chemical data were obtained using the Gaussian09 ^31^ suite of programs and the geometries visualized using GaussView6.^32^

## 3 Results and Discussion

### 3.1 CEMB Experiments for ASCF_3_

In the gas phase experiment, the molecules were exposed to electrons with well-defined and controlled energies to investigate how the ASCF_3_ molecule dissociates upon electron attachment within the energy range of 0–15 eV. In addition to the molecular anion of ASCF_3_, we observed ten fragment anions within the detection range. The anion efficiency curves, which demonstrate characteristic resonance energies, are presented in Figures 1 and 2.

**Figure 1:**
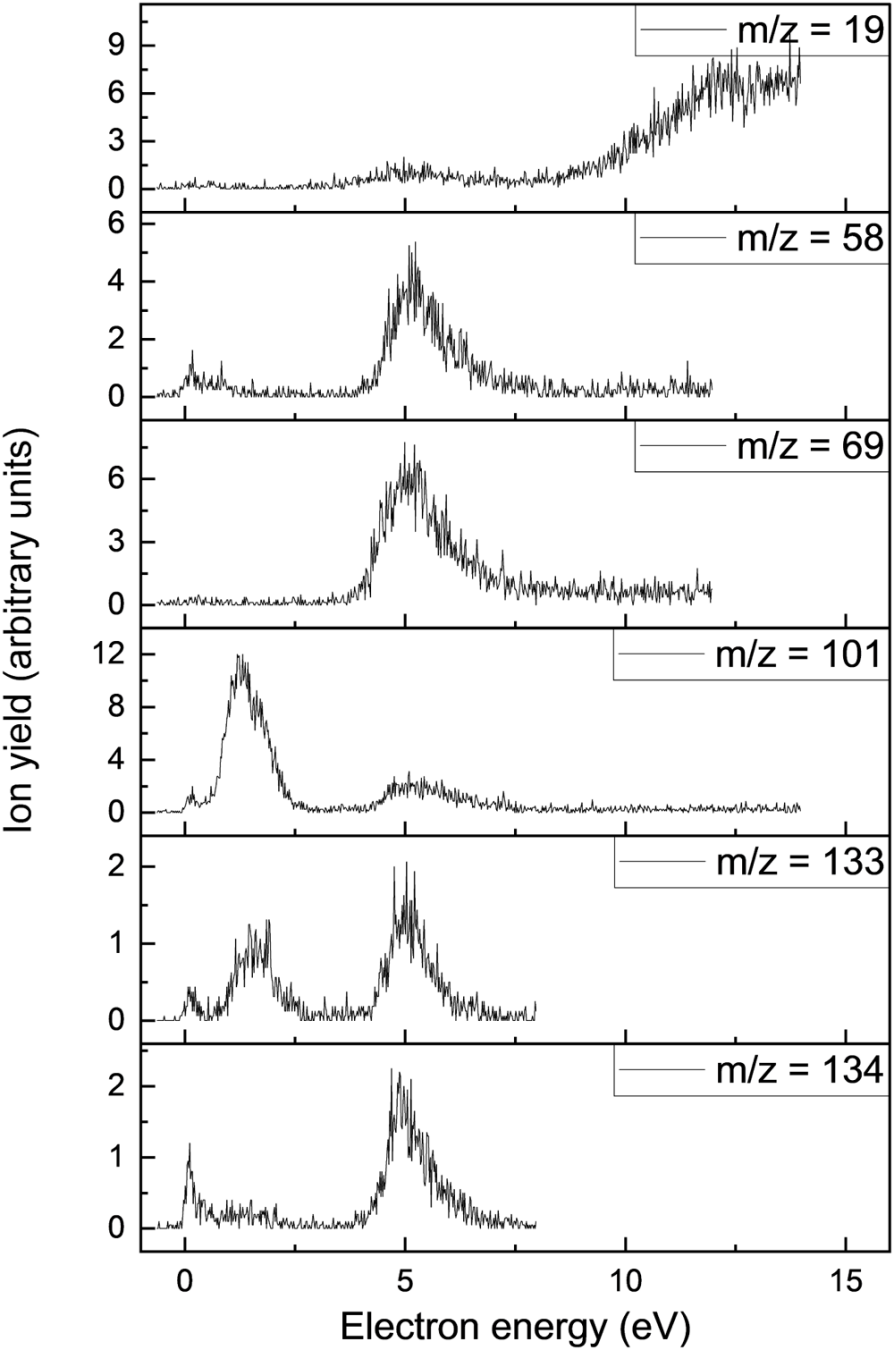
Anion efficiency curves of the fragment anions formed upon electron attachment to ASCF_3_: *m*/*z* = 19, *m*/*z* = 58, *m*/*z* = 69, *m*/*z* = 101, *m*/*z* = 133, and *m*/*z* = 134. The black line corresponds to the measured ion yield.

**Figure 2:**
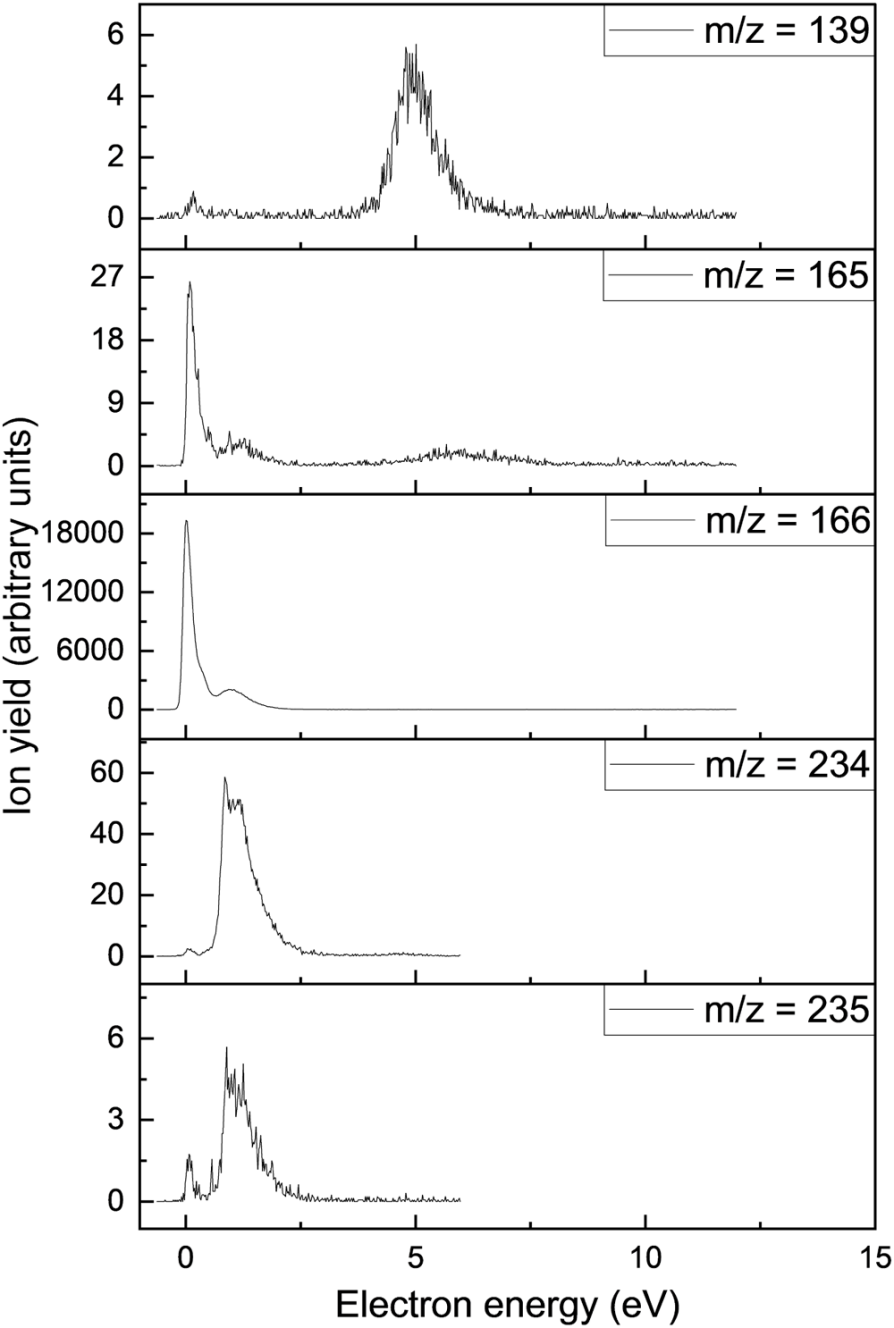
Anion efficiency curves of the fragment anions formed upon electron attachment to ASCF_3_: *m*/*z* = 139, *m*/*z* = 165, *m*/*z* = 166, *m*/*z* = 234, and *m*/*z* = 235. The black line corresponds to the measured ion yield.

The purpose of the present DEA study was not to directly reproduce physiological conditions, but rather to investigate the intrinsic electron-capture properties of the isolated molecule and to identify the transient negative ion states and fragmentation pathways that become accessible upon electron attachment. Gas-phase experiments provide fundamental information on the energetics and mechanisms of electron-induced bond cleavage without the complicating effects of solvation, protonation, and intermolecular interactions. It should also be noted that ionizing radiation initially produces a broad distribution of secondary electrons, including electrons with energies extending well above several electronvolts, before thermalization occurs. Consequently, the 0–15 eV energy range investigated in this work encompasses not only near-thermal electrons but also higher-energy secondary electrons that may be present during the early stages of radiation track formation. The resonances identified in this energy range therefore provide insight into potential electron-induced damage pathways that may occur before complete electron solvation. At the same time, we acknowledge that electron attachment processes in aqueous media can differ substantially from those observed in the gas phase because solvation and protonation may alter both the energetics and the reaction pathways. Therefore, the present results should be regarded as a characterization of the intrinsic DEA behavior of the molecule, while extrapolation to biological environments requires additional experimental and theoretical studies that explicitly account for solvent effects.

The experimentally determined threshold energy for each fragment anion, derived using a previously established method,^20^ is listed in Table 1 and compared with computationally obtained thresholds.

**Table 1:** A Summary of the Observed Fragment Anions is Provided, Detailing Their Masses, Structural Assignments, and Corresponding Peak Values on the Anion Efficiency Curves, along with the Experimental and Calculated Threshold Energies (ΔE_0_)

		Maxima of peak positions (eV)				Threshold (eV)	
Mass	Anion				Max intensivity (a.u.)	Expt (356 K)	Calcd (ΔE_0_)
19	[F]^−^	5.2	13.0		6.9	2.75	1.97
58	a[SCN]^−^	0.1	0.6	5.3	3.5	0.0	–0.57
	b[SCN]^−^						0.30
	c[SCN]^−^						3.74
69	[CF_3_]^−^	5.2			5.6	3.6	0.97
101	[SCF_3_]^−^	0.1^b^	1.4	4.9	10.5	0.0^b^, 0.6	0.50
133	a[ASCF_3_–HSCF_3_]^●–^	0.1^b^	1.6	5.0	1.3	0.0^b^, 0.7	0.80
	b[ASCF_3_–HSCF_3_]^●–^						1.70
	c[ASCF_3_–HSCF_3_]^●–^						2.20
134	[ASCF_3_–SCF_3_]^−^	0.1^b^	1.1^b^	5.0	1.6	0.0^b^, 4.1	1.60
139	a[ASCF_3_–HCN-CF_3_]^−^	0.2^b^	5.0		4.4	0.0^b^, 4.1	4.19
	b[ASCF_3_–HCN-CF_3_]^−^						4.22
	c[ASCF_3_–HCN-CF_3_]^−^						4.77
	d[ASCF_3_–HCN-CF_3_]^−^						4.82
	e[ASCF_3_–HCN-CF_3_]^−^						6.36
	a[ASCF_3_–HNC–CF_3_]^−^						4.75
	b[ASCF_3_–HNC–CF_3_]^−^						4.78
	c[ASCF_3_–HNC–CF_3_]^−^						5.32
	d[ASCF_3_–HNC–CF_3_]^−^						5.38
	e[ASCF_3_–HNC–CF_3_]^−^						6.92
165	a[ASCF_3_–HCF_3_]^●–^	5.8			1.8	4.1	–1.11
	b[ASCF_3_–HCF_3_]^●–^						–1.01
	c[ASCF_3_–HCF_3_]^●–^						–0.22
166	[ASCF_3_–CF_3_]^−^	0.1	0.3	1.2	19600.0	0.0	–0.24
234	a[ASCF_3_–H]^−^	0.1^b^	0.8	1.2	58.0	0.0,^b^ 0.7	0.47
	b[ASCF_3_–H]^−^						1.36
	c[ASCF_3_–H]^−^						3.34
235	ASCF_3_^●–^	∼0			1.3	0.0	–0.74^a^

a Calculated as the difference between the electronic energy corrected for zero-point energies of the neutral and product complex, since the ASCF_3_ anion is geometrically unstable (see the text).

b Threshold and therefore the corresponding peak not supported by theory (see the text).

AEA of the ASCF_3_ molecule (the difference between Gibbs Free Energies for the optimized (SCF_3_^–^···[ASCF_3_–SCF_3_]^●^) molecular complex and the neutral ASCF_3_ molecule) is positive and calculated to be 0.69 eV at the M06-2X^23^/aug-cc-pVTZ^33^ level. DFT optimization indicates that, after electron attachment, the structure of the ASCF_3_ anion is not geometrically stable and undergoes dissociation into an anionic and radical fragment. When the S–C bond length is constrained at the value of 1.82 Å (the S-CF_3_ bond length in the neutral form of ASCF_3_), the electron affinity remains positive and amounts to 0.35 eV.

Dissociative electron attachment (DEA) to ASCF_3_ leads to the formation of ten fragment anions as indicated by the CEMB experiment, few of them resulting from the cleavage of bonds within the adenine moiety. Figure 3 depicts different dissociation pathways leading to the observed anions.

**Figure 3:**
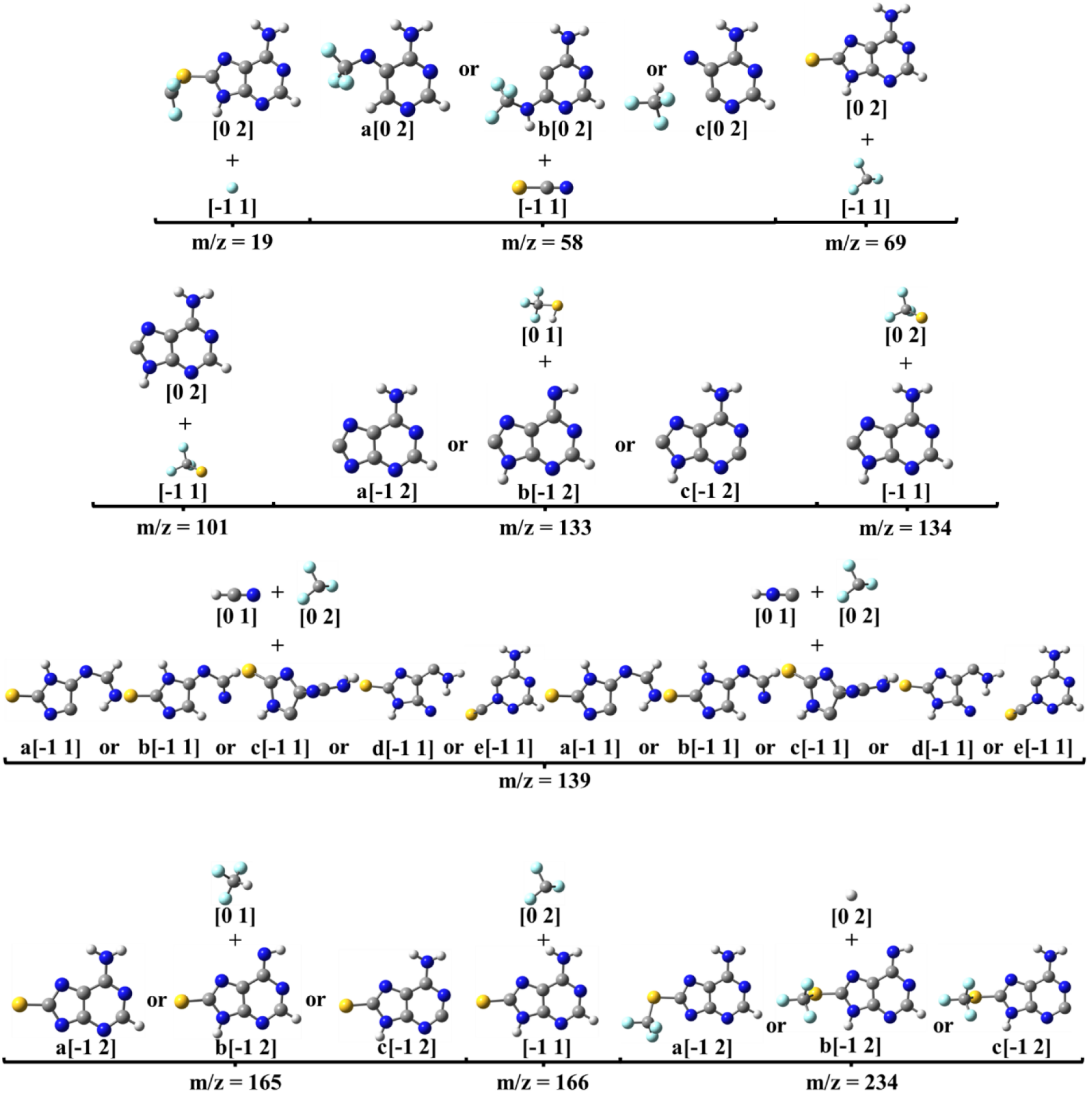
Dissociation products of the ASCF_3_ molecule upon low-energy electron attachment. The experimentally observed fragments are identified by their *m*/*z* values: 19, 58, 69, 101, 133, 134, 139, 165, 166, and 234. For each structure, charge and multiplicity are indicated in brackets. The following color codes were used to indicate atoms: white for H, gray for C, blue for N, yellow for S, and cyan for F.

The lightest observed anion (see Figure 3, *m*/*z* = 19), with an *m*/*z* ratio of 19, originates from the cleavage of the C–F bond. This reaction can be described as follows:

1 $$e+ASCF_{3}\to ASC{F_{3}}^{●-}\to F^{-}+{\left[ASCF_{3}‐F\right]}^{●}$$

The thermodynamic threshold calculated at the M06–2X/aug-cc-pVTZ level amounts to 1.97, while the CEMB experiment gives 2.75 eV for the occurrence of this anion (see Table 1 and Figure 1). This deviation indicates that the fragment anion cannot be formed near the thermodynamic threshold due to the resonance nature of DEA. Moreover, the anion efficiency curve shows also raising intensity above about 8 eV, which may be indicating dipolar dissociation upon electron impact.^34^

The subsequent anion of *m*/*z* equal to 58 (see Figure 3, *m*/*z* 58 a, b or c) corresponds to the cleavage of the purine ring along with the formation of the SCN^–^ anion. The lowest calculated energy for this DEA reaction pathway is −0.57 eV, which is in good accordance with the experimental threshold of 0 eV (see Table 1 and Figure 1).

2 $$e+ASCF_{3}\to ASC{F_{3}}^{●-}\to SCN^{-}+{\left[ASCF_{3}‐SCN\right]}^{●}$$

A further anion detected in the CEMB experiment has an *m*/*z* of 69, originating from the dissociation of the S–CF_3_ bond (see Figure 3, *m*/*z* = 69). The energy for this dissociation (3) is calculated to be 0.97 eV, while its CEMB value is equal to 3.6 eV (see Table 1).

3 $$e+ASCF_{3}\to ASC{F_{3}}^{●-}\to {CF_{3}}^{●}+{\left[ASCF_{3}‐CF_{3}\right]}^{●}$$

The anion with an *m*/*z* of 101 has been assigned to SCF_3_^–^ (see Figure 3, *m*/*z* = 101), arising from the cleavage of the C–SCF_3_ bond, which can be described by the following reaction equation:

4 $$e+ASCF_{3}\to ASC{F_{3}}^{●-}\to {SCF}_{3}^{-}+{\left[ASCF_{3}‐SCF_{3}\right]}^{●}$$

The computational threshold of 0.5 eV agrees well with the experimental value of 0.6 eV, corresponding to the onset of the second peak with a maximum at 1.4 eV (Figure 1 and Table 1). The small peak with a maximum at 0.1 eV (Figure 1 and Table 1) represents a typical contribution from electron attachment to vibrationally excited neutral molecules or an impurity.

Two additional anions detected in the CEMB experiment exhibit structural similarity. The first, with an *m*/*z* ratio of 133, results from the detachment of an H–SCF_3_ fragment (the hydrogen atom can originate from the N9, C2, or NH_2_ sites, see Figure 3, *m*/*z* = 133, a, b, or c), with the lowest-energy dissociation pathway (eq 5) calculated at 0.80 eV (see Table 1) with the reasonable agreement with the experimental onset (0.7 eV) of the second peak with the maximum at 1.6 eV (Figure 1). The resonance at 0.1 eV can be ascribed, similarly as for *m*/*z* 101 anion, to electron attachment to vibrationally excited neutral or an impurity. The second, with an *m*/*z* ratio of 134, originates from the dissociation of the C8–SCF_3_ bond (see Figure 3, *m*/*z* = 134), leading to the formation of an anion within the purine moiety. The reaction energetics for this pathway (eq 6) have been determined as 1.60 eV (see Table 1).

5 $$e+ASCF_{3}\to ASC{F_{3}}^{●-}\to {\left[ASCF_{3}‐HSCF_{3}\right]}^{●-}+HSCF_{3}$$

6 $$e+ASCF_{3}\to ASC{F_{3}}^{●-}\to {\left[ASCF_{3}‐SCF_{3}\right]}^{-}+SC{F_{3}}^{●}$$

Another anion detected during the CEMB experiment has an *m*/*z* ratio of 139. In this case, ten possible dissociation pathways (eq 7) were proposed (see Figure 3). The lowest-energy dissociation pathway (4.19 eV, see Table 1) involves the cleavage of the S–CF_3_ bond alongside purine ring rupture, with the simultaneous release of hydrogen cyanide (HCN) (see Figure 3, *m*/*z* = 139). One may consider the small 0 eV contribution in the anion efficiency curve (see Figure 2) as an artifact, since the main resonance is too high in energy, assuming that the 0.2 and 5 eV resonances (Table 1) differ due to vibrational excitation of the neutral. Thus, the calculated threshold of 4.19 eV is near to the experimental value of 4.1 eV for the peak with maximum at 5.0 eV (see Figure 2).

7 $$e+ASCF_{3}\to ASC{F_{3}}^{●-}\to {[ASCF_{3}‐HCN‐CF_{3}]}^{-}+C{F_{3}}^{●}+HCN$$

Two subsequent anions seem structurally similar. The first one (eq 8), with an *m*/*z* ratio of 165, may arise from the dissociation of the S–CF_3_ bond along with the C–H bond cleavage (the hydrogen atom may come from N9, C2 atoms, or NH_2_ group, see Figure 3a–c) and formation of neutral trifluoromethane. The reaction energetics for this process were determined to be −1.11 eV (see Table 1). However, experimentally we only find a weak peak at about 5.8 eV (the yields at low-energy can be ascribed to [ASCF_3_–CF_3_]^−^ due to limited mass resolution), which may indicate separation of H + CF_3_. Indeed, the calculated threshold spans a range of 3.4–4.3 eV depending on the adenine site from which the hydrogen atom is released, which agrees well with the measured value of 4.1 eV (see Table 1). The second one (eq 9), with an *m*/*z* ratio of 166, is the most intense anion observed in the CEMB experiment and arises from S-CF_3_ dissociation (see Figure 3, *m*/*z* = 166), with an associated reaction energy of −0.24 eV (see Table 1) and the experimental threshold of 0 eV (see Figure 1 and Table 1) in agreement with the DFT model (see Table 1).

8 $$e+ASCF_{3}\to ASC{F_{3}}^{●-}\to {[ASCF_{3}‐HCF_{3}]}^{●-}+HCF_{3}$$

9 $$e+ASCF_{3}\to ASC{F_{3}}^{●-}\to {[ASCF_{3}‐CF_{3}]}^{-}+C{F_{3}}^{●}$$

The second heaviest anion with the *m*/*z* of 234 originates from a simple dissociation of the C–H bond (the hydrogen atom may come from N9, C2 atoms, or NH_2_ group, see Figure 3, a, b, or c). The threshold energy for this reaction was determined to be 0.47 eV, and it is slightly lower than the experimental value of 0.7 eV (see Table 1).

10 $$e+ASCF_{3}\to ASC{F_{3}}^{●-}\to {[ASCF_{3}‐H]}^{-}+H^{●}$$

The heaviest mass (eq 11) detected in the CEMB experiment corresponds to that of the parent anion with the *m*/*z* ratio of 235. The reaction of this electron attachment process can be expressed as

11 $$e+ASCF_{3}\to ASC{F_{3}}^{●-}$$

The anion efficiency curve at *m*/*z* 235 and *m*/*z* 234 are very similar, expect the presence of the enhanced peak at about 0 eV in the yield at *m*/*z* 235. We ascribe only this zero eV peak to the formation of the parent anion via reaction 11 while the other yield at higher electron energy can be assigned to the isotope of [ASCF_3_–H]^−^. The calculated threshold for reaction 11 equals to −0.74 eV. This indicates a positive electron affinity of ASCF_3_ which agrees with the detection of the parent anion in the present experiment.

### 3.2 Radiolysis of ASCF_3_ in an Aqueous Solution

The above-discussed results concerning the gas-phase experimental studies remain in very good agreement with the quantum chemical calculations. The predominant anion formed during the degradation of ASCF_3_ upon electron attachment has a mass-to-charge ratio (*m*/*z*) of 166. This observation is consistent with results obtained for another modified adenine derivative—ASCH_3_.^8^ The high intensity of this ion may suggest that the same dissociation pathway is also dominant in aqueous solution.

To investigate whether the proposed adenine derivative undergoes dissociative electron attachment (DEA) also in the aqueous phase, we performed radiolysis experiments under reducing conditions that mimic the hypoxic environment observed in many cancers.^35^ A detailed description of the experimental protocol is provided in the Methods section.

The results of the radiolytic experiments differed markedly from those obtained from the gas-phase. In particular, stationary radiolysis of an aqueous ASCF_3_ solution does not result in the formation of the AS anion and the CF_3_ radical, which would be expected if reaction (6) were the dominated DEA pathway in water. Instead, the HPLC chromatogram reveals the formation of a single major product (see Figure 4, red trace) with a retention time of 2.98 min and an ion at *m*/*z* 214.0178 in the HRMS (ESI^–^) spectrum (see Figure S7). The observed ion corresponds to the [M–H]^−^ species, where M denotes the neutral molecular product formed after HF elimination (molecular mass 215 Da; [ASCF_3_–HF]). Thus, the detected ion at *m*/*z* 214 results from deprotonation of this neutral product in the negative ionization mode (calculated *m*/*z* for [M–H]^−^ = 213.9999; see Figures S7 and S8). This observation suggests that the primary DEA pathway in solution does not end up with the S–C bond cleavage in contrast to the gas-phase mechanism (eq 9).

**Figure 4:**
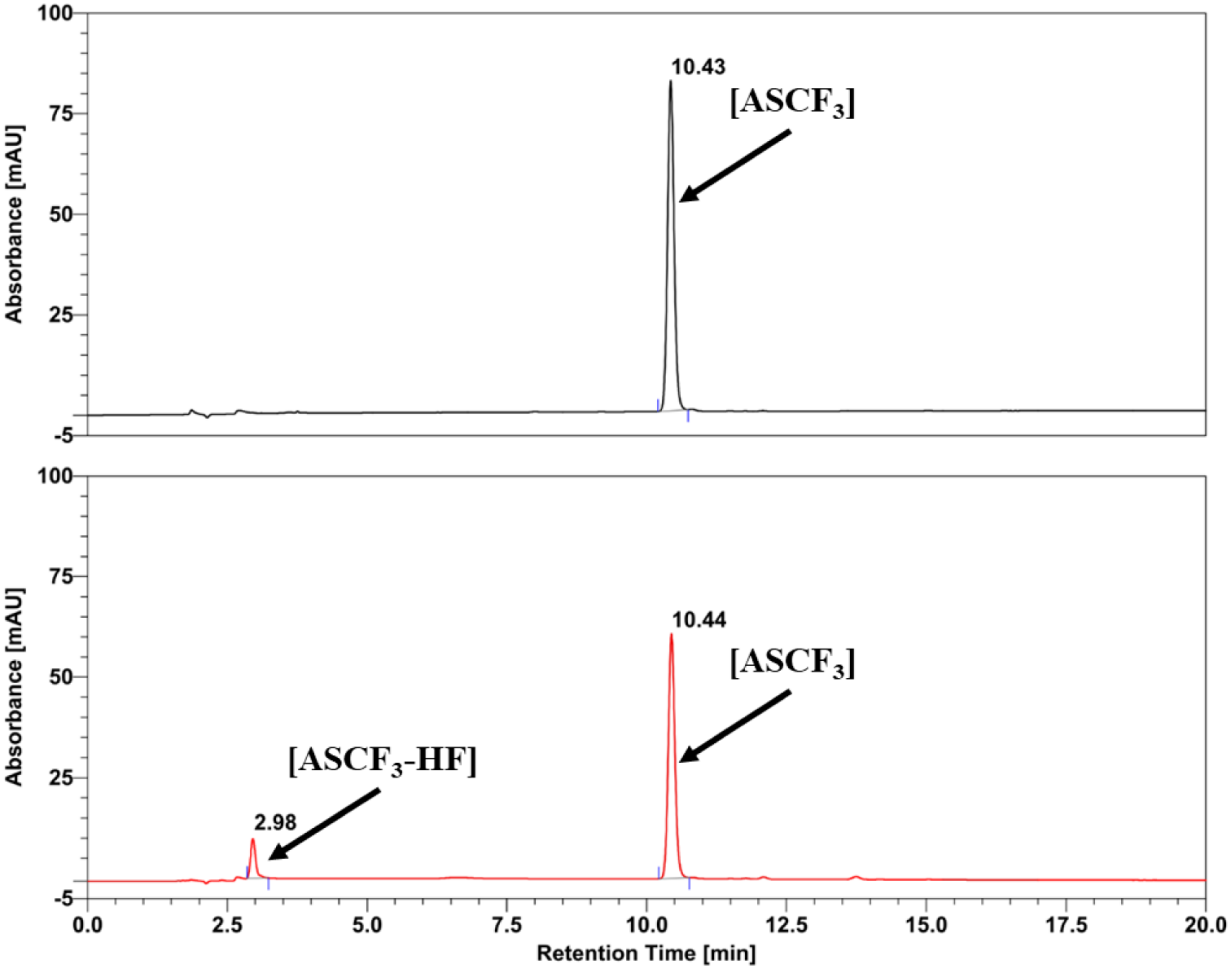
HPLC analysis of ASCF_3_ in water before (black line) and after irradiation with a dose equal to 500 Gy (red line).

To explain the discrepancy between the gas- and liquid-phase results, we carried out DFT modeling in an aqueous environment at the M06-2X^23^/6-31++G­(d,p)^26^ level of theory, assuming the proper protonation state of ASCF_3_. Indeed, in water at pH 7, ASCF_3_ occurs in a monoprotonated form (protonated at N1, [ASCF_3_+H]^+^), as indicated by its computed *pK*_*a*_ of 8.9. ASCH_3_, which has a measured *pK*_*a*_ of 8.83, very similar to that of ASCF_3_, is completely protonated at neutral pH, as shown by potentiometric titration.^8^ Thus, the [ASCF_3_–HF] product of DEA (see Figure 5) might form due to C–F bond cleavage in the [ASCF_3_+H]^●^ radical (the latter is formed upon electron attachment to the monoprotonated [ASCF_3_+H]^+^ cation), yielding an [ASCF_3_+H-HF]^●^ radical and a HF molecule.

**Figure 5:**
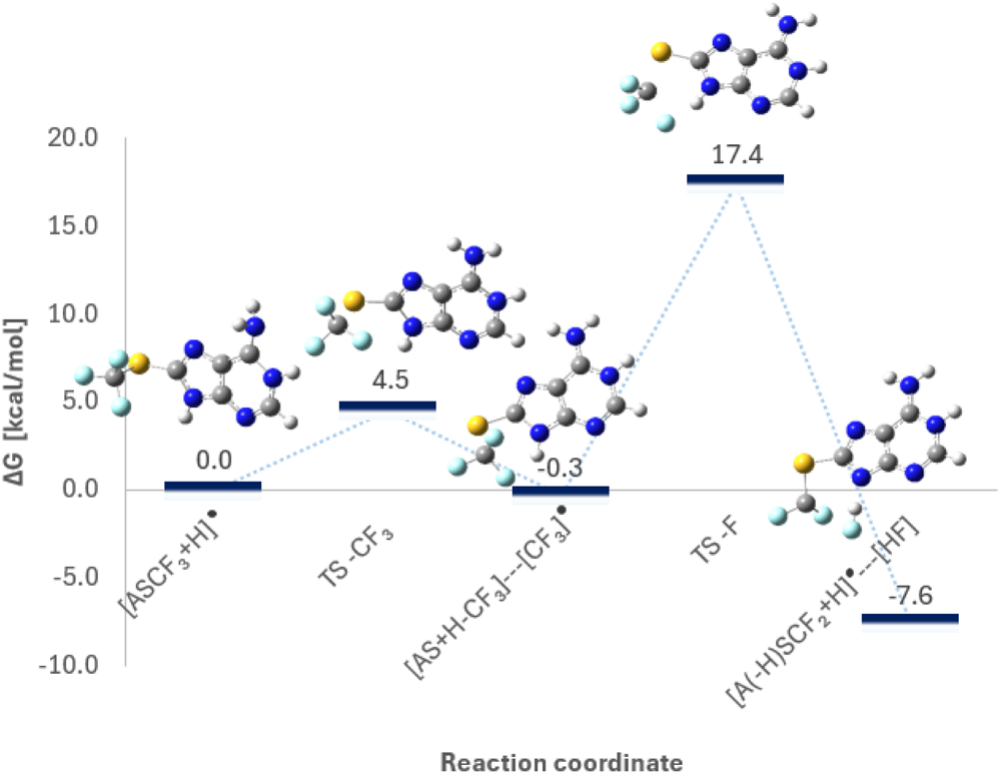
Free energy changes on the DEA pathway of the N1-protonated ASCF_3_ cation ([ASCF_3_+H]^+^) in water (PCM). The AEA (not shown on the profile for clarity) is 2.9 eV.

In light of this apparent inconsistency—namely, that a chemical reaction is expected to proceed along the lowest-barrier pathway, whereas no stable radiolysis products derived from the CF_3_ radical and AS^–^ anion were detected—we examined the dissociation profile of the [ASCF_3_+H]^●^ radical. Already in the gas phase, C–S bond cleavage is associated with a substantial activation energy (Δ*G* ^‡^ = 18.3 kcal/mol; see Figure S9) consistent with the general observation that dissociation barriers are significantly elevated in protonated electron adducts.^36,37^ In contrast, direct fluoride anion elimination in the gas phase involves much higher barrier (Δ*G* ^‡^ ca. 44.4 kcal/mol; see Figure S9). However, this process becomes considerably more favorable when it proceeds via stepwise mechanism. Initially, S–C bond cleavage occurs with Δ*G* ^‡^ = 18.3 kcal/mol, followed by a concerted elimination of F^–^ from the CF_3_ radical and H^+^ from the N9 site of the adenine moiety (i.e., elimination of HF) accompanied by reformation of the S–C bond between adenine and the remaining −CF_2_ fragment (Δ*G* ^‡^ = 18.0 kcal/mol; see Figure S9).

In aqueous solution, as simulated by polarizable continuum model (PCM),^28^ these barriers are substantially reduced relative to the gas phase (Figure 5). Specifically, separation of the [ASCF_3_+H]^●^ radical from the CF_3_ fragment proceeds over barrier of only 4.5 kcal/mol (Δ*G*_r_ = −0.3 kcal/mol). The subsequent step—concerted elimination of HF and reattachment of the CF_2_ fragment, leading to a complex composed of the [A­(−H)­SCF_2_+H]^●^ radical and an HF molecule—is characterized by Δ*G* ^‡^ = 17.7 kcal/mol and Δ*G*_r_ = −7.3 kcal/mol. These results indicate that the overall process is thermodynamically and kinetically accessible under ambient conditions.

Electron transfer between the [A­(−H)­SCF_2_+H]^●^ radical and the tBuO^●^ radical (see eq 12)—the latter formed via scavenging of hydroxyl radicals by tert-butanol—followed by cyclization (see eq 13 and Figure 6), is characterized by a negative reaction free energy (Δ*G*_r_ = −8.0 kcal mol^–1^ and a relatively low kinetic barrier (Δ*G* = 13.7 kcal mol^–1^; see Figure 6). In this cyclic form, the DEA product most likely corresponds to the species detected in the LC–MS experiment. Thus, the mechanism outlined above provides a consistent explanation of DEA in aqueous solution.

12 $${[A(‐H)SCF_{2}+H]}^{●}+tBuO^{●}\to {[A(‐H)SCF_{2}+H]}^{+}+tBuO^{-}$$

13 $${[A(‐H)SCF_{2}+H]}^{+}\to Cyclic{[A(‐H)SCF_{2}+H]}^{+}$$

**Figure 6:**
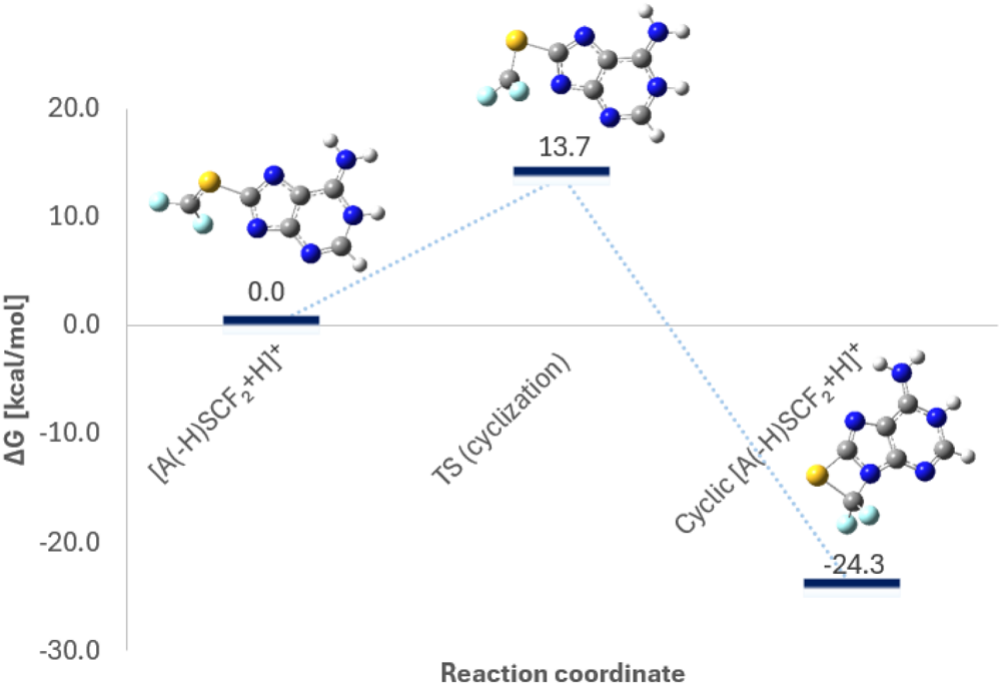
[A­(−H)­SCF_2_+H]^+^ cyclization process (Gibbs free energy profile).

It is worth emphasizing that the [A­(−H)­SCF_2_+H]^●^ radical has sufficient hydrogen-atom affinity to damage DNA by abstracting one of its deoxyribose hydrogens. The Gibbs free energy for hydrogen-atom transfer from the individual deoxyribose sites C1′, C2′, C3′, C4′, and C5′ to the CF_2_ carbon of [A­(−H)­SCF_2_+H]^●^ amounts to −5.8, −1.0, −5.5, −8.1, and −5.1 kcal mol^–1^, respectively, indicating that the C4′ site is the most sensitive toward hydrogen abstraction.^38^ This favorable thermodynamics thus suggests that the ASCF_3_ derivative of adenine should enhance ionizing-radiation-induced cellular DNA damage.

## 4 Summary

The presented results demonstrate that, in addition to modified pyrimidines, suitably functionalized purine derivatives can also act as efficient radiosensitizers. By combining complementary experimental approaches with quantum-chemical modeling, this work provides a framework for the rational development of new radiosensitizing agents aimed at enhancing the therapeutic efficacy of radiotherapy, particularly in hypoxic tumors. These findings advance the search for molecules that harness dissociative electron attachment (DEA) and offer practical guidelines for the molecular design of clinically relevant radiosensitizers.

Thus, we report 8-(trifluoromethyl)­thioadenine (ASCF_3_) as a novel purine-based candidate radiosensitizer designed to exploit dissociative electron attachment (DEA) under hypoxic conditions. Combining crossed electron–molecule beam experiments with DFT calculations, we show that ASCF_3_ exhibits a high propensity for low-energy electron attachment in the gas phase, yielding ten fragment anions with [ASCF_3_–CF_3_]^−^ (*m*/*z* 166) as the dominant product, consistent with efficient S-CF_3_ bond cleavage. In contrast, stationary radiolysis of ASCF_3_ in aqueous solution under reducing, hypoxia-mimetic conditions produces a single major product assigned to an [ASCF_3_–HF] species, indicating a solution-phase DEA pathway that does not proceed via simple S–C bond scission and CF_3_ radical release. PCM-based DFT calculations, accounting for the monoprotonated form of ASCF_3_ at neutral pH, support a stepwise mechanism involving initial S–CF_3_ bond rupture in the [ASCF_3_+H]^●^ radical, followed by concerted HF elimination and S–C bond reformation to a CF_2_ fragment, ultimately yielding a cyclic [A­(−H)­SCF_2_+H]^+^ product in agreement with LC–MS data. The resulting [A­(−H)­SCF_2_+H]^●^ radical exhibits favorable hydrogen-atom abstraction thermodynamics toward deoxyribose sites, suggesting that the corresponding nucleoside could efficiently promote DNA strand breaks. These findings demonstrate that C8-modified purines bearing an −SCF_3_ group form a promising platform for DEA-based radiosensitizer design and emphasize that solvent and protonation effects are crucial for correctly identifying the operative degradation pathways.

## Supplementary Material



## Notes

The ASCF3_SI.docx contains additional figures mentioned in the manuscript, XYZ geometries of all stationary points are deposited in the zenodo.org repository, DOI: 10.5281/zenodo.17699238. The CEMB data can be found at 10.5281/zenodo.16793430 (PDF)
