# Supplementary material for: Dissociative Electron Attachment to 8‑(Trifluoromethyl)thioadenine: A Potential Radiosensitizer
Source: J Phys Chem B. 2026 Jul 20;130(30):7578–87. doi: 10.1021/acs.jpcb.6c03946 (PMC13430633; doi:10.1021/acs.jpcb.6c03946)
Supplement: Supplementary file 1 [file jp6c03946_si_001.pdf]

# Supporting Information

# Dissociative Electron Attachment to 8-(trifluoromethyl)thioadenine: A Potential Radiosensitizer

Adrian Szczyrba,[a] Magdalena Datta,[a,b] Samanta Makurat,[a] Jiakuan Chen,[c,d] Karol Biernacki,[e] Sebastian Demkowicz,[e] Stephan Denifl,[c,d*] Janusz Rak[a*]

[a] Laboratory of Biological Sensitizers, Department of Physical Chemistry, Faculty of Chemistry, University of Gdańsk, Wita Stwosza 63, 80-308 Gdańsk, Poland

[b] Department of Inorganic Chemistry, Faculty of Chemistry, Gdańsk University of Technology, Narutowicza 11/12, 80-233 Gdańsk, Poland

[c] Institut für Ionenphysik und Angewandte Physik, Universität Innsbruck, Technikerstraße 25, A-6020 Innsbruck, Austria

[d] Center for Molecular Biosciences Innsbruck, Universität Innsbruck, Technikerstraße 25, A-6020 Innsbruck, Austria

[e] Department of Organic Chemistry, Faculty of Chemistry, Gdańsk University of Technology, Narutowicza 11/12, 80-233 Gdańsk, Poland

*Correspondence: stephan.denifl@uibk.ac.at, janusz.rak@ug.edu.pl

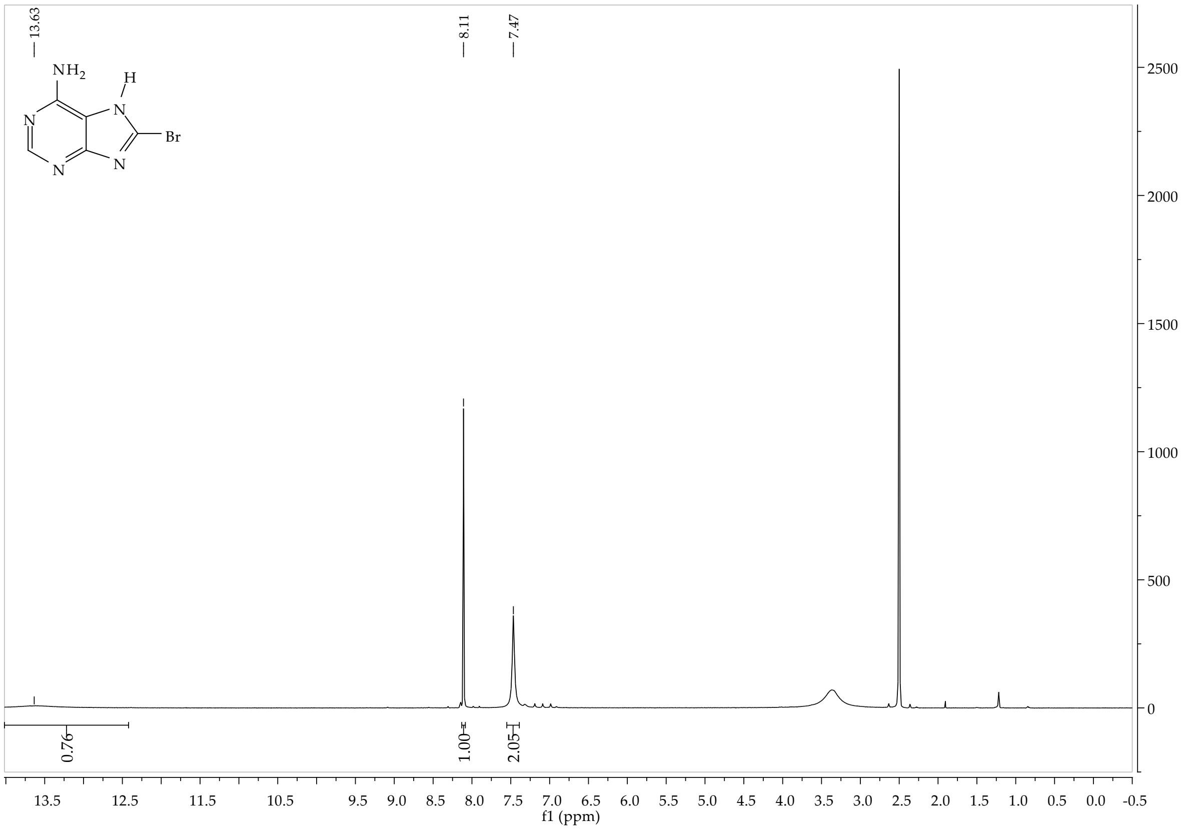


**Figure S1.** $^1$H NMR spectra of 8-bromoadenine (500 MHz, DMSO-$d_6$) $\delta_H$ 13.63 (1H, bs, NH) 8.11 (1H, s, CH), 7.47 (2H, bs, $NH_2$).

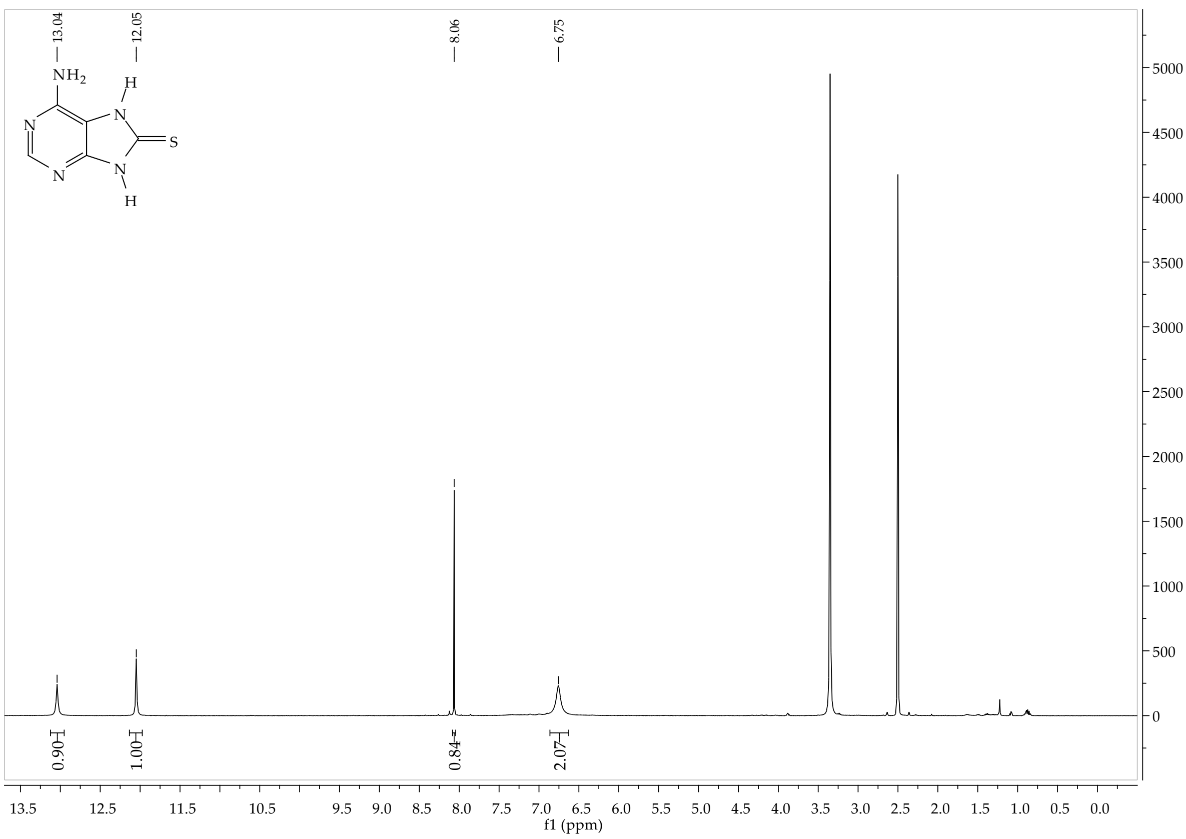


**Figure S2.** $^{1}$H NMR spectra of 6-amino-7,9-dihydro-8*H*-purine-8-thione (500 MHz, DMSO-$d_6$, Figure S2) $\delta_H$ 13.04 (1H, s, NH), 12.05 (1H, bs, NH), 8.06 (1H, s, CH) 6.75 (2H, bs, $NH_2$).

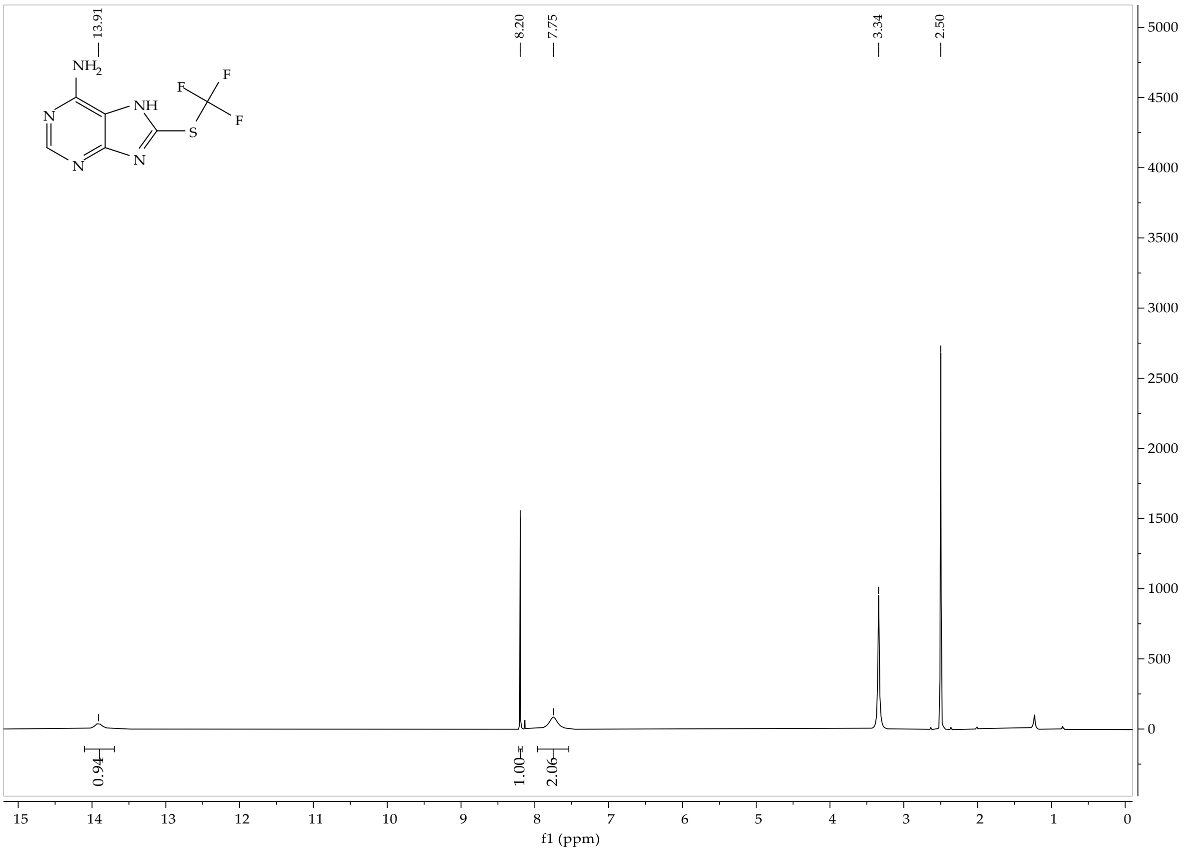


**Figure S3.** $^{1}$H NMR spectra of 8-(trifluoromethyl)thioadenine (DMSO-$_{d6}$, 500MHz), 13.91 (1H, bs, NH), 8.20 (1H, s, CH), 7.75 (2H, bs, $NH_2$),

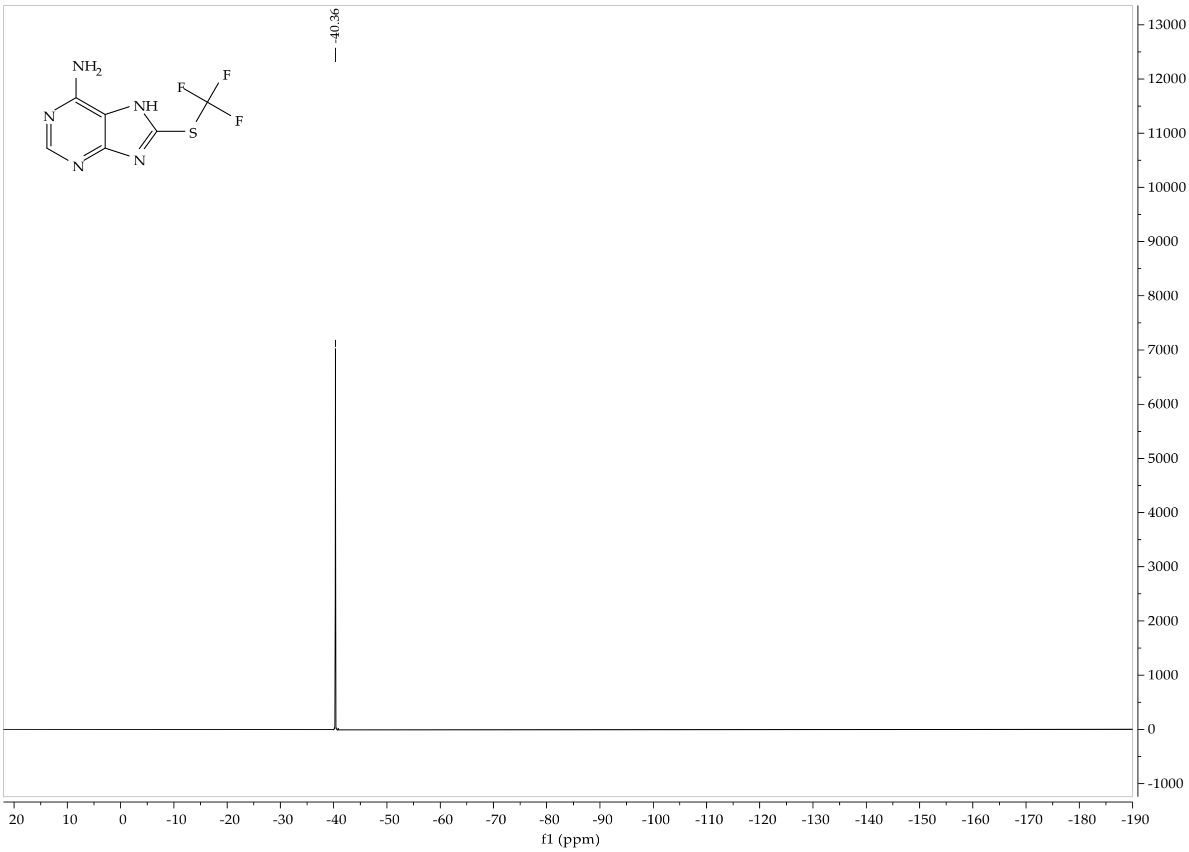


**Figure S4.** $^{19}$F NMR spectra of 8-(trifluoromethyl)thioadenine ($^{19}$F NMR (DMSO-$_{d6}$, 470MHz), -40.36).

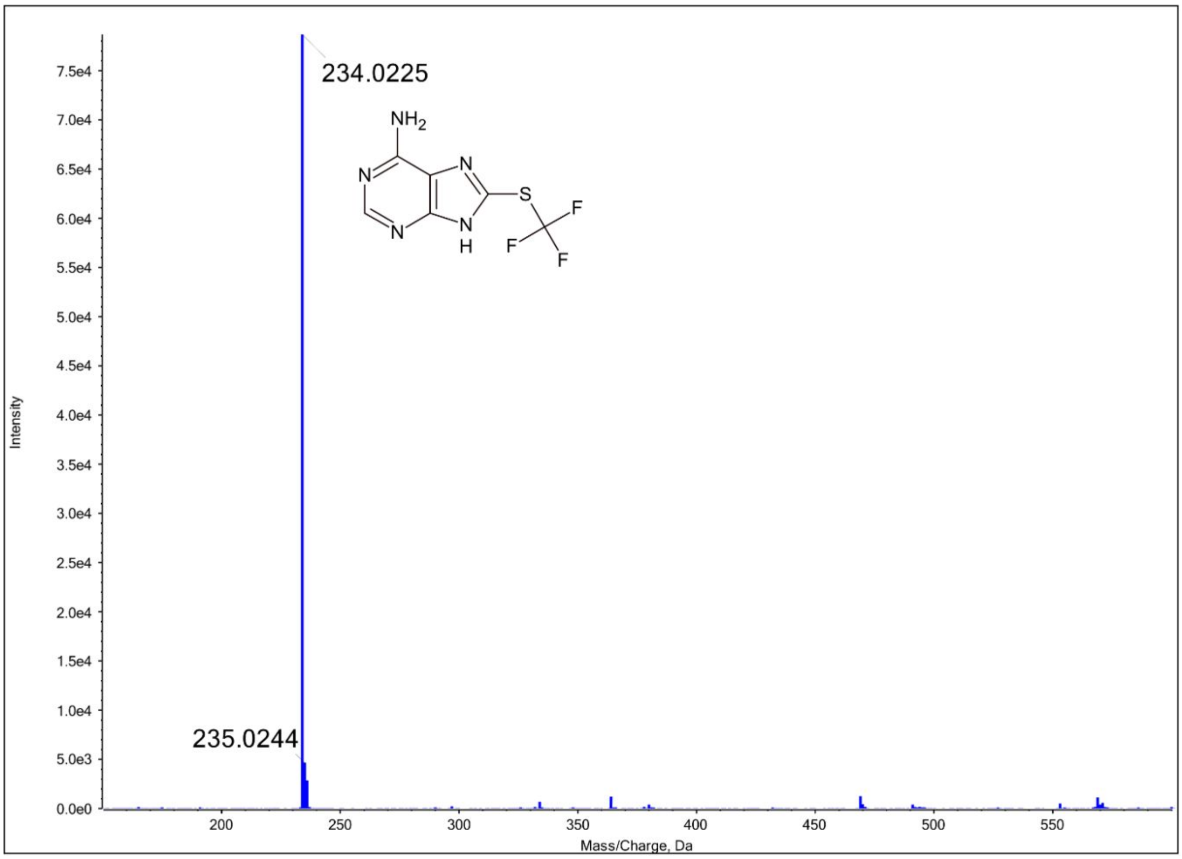


**Figure S5.** HRMS spectrum of 8-(trifluoromethyl)thioadenine registered in negative ionization mode. HRMS $(ESI)^-$ m/z: $[M-H]^-$ calcd for C6H3N5SF3 234.0061; found 234.0225.

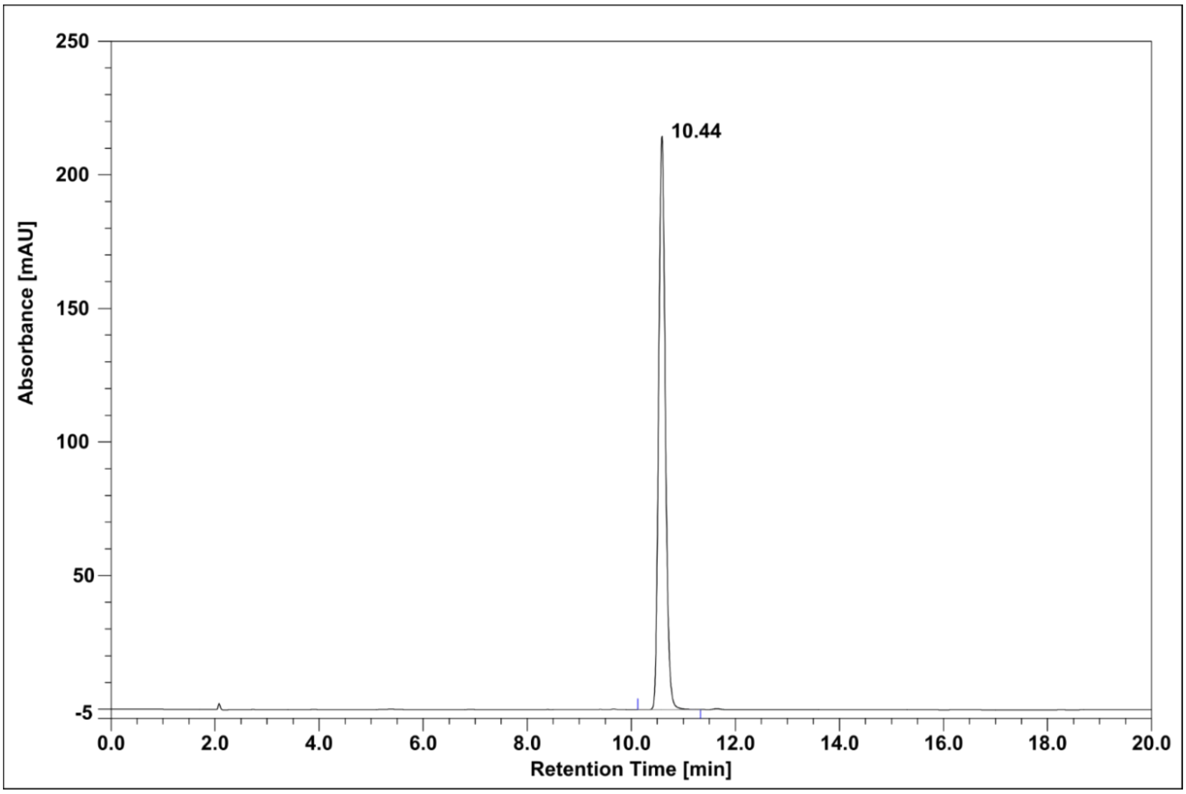


**Figure S6.** HPLC of 8-(trifluoromethyl)thioadenine (purity = 98%).

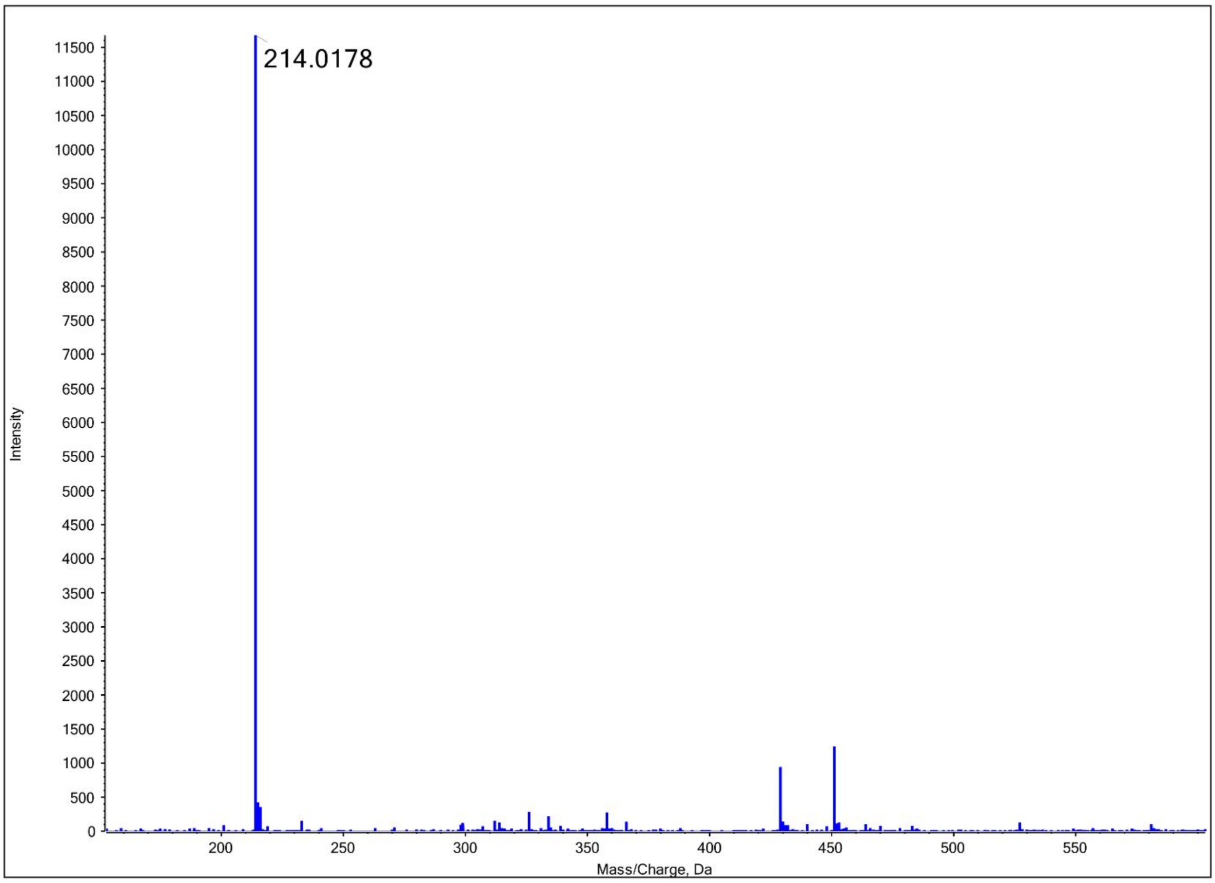


**Figure S7.** MS spectrum of radiolysis product corresponding to the peak at 2.98 min on a HPLC chromatogram (see Figure 4). HRMS (ESI)$^-$ m/z: [M−H]$^-$ calcd for C6H2N5SF2 213.9999 , found 214.0178.

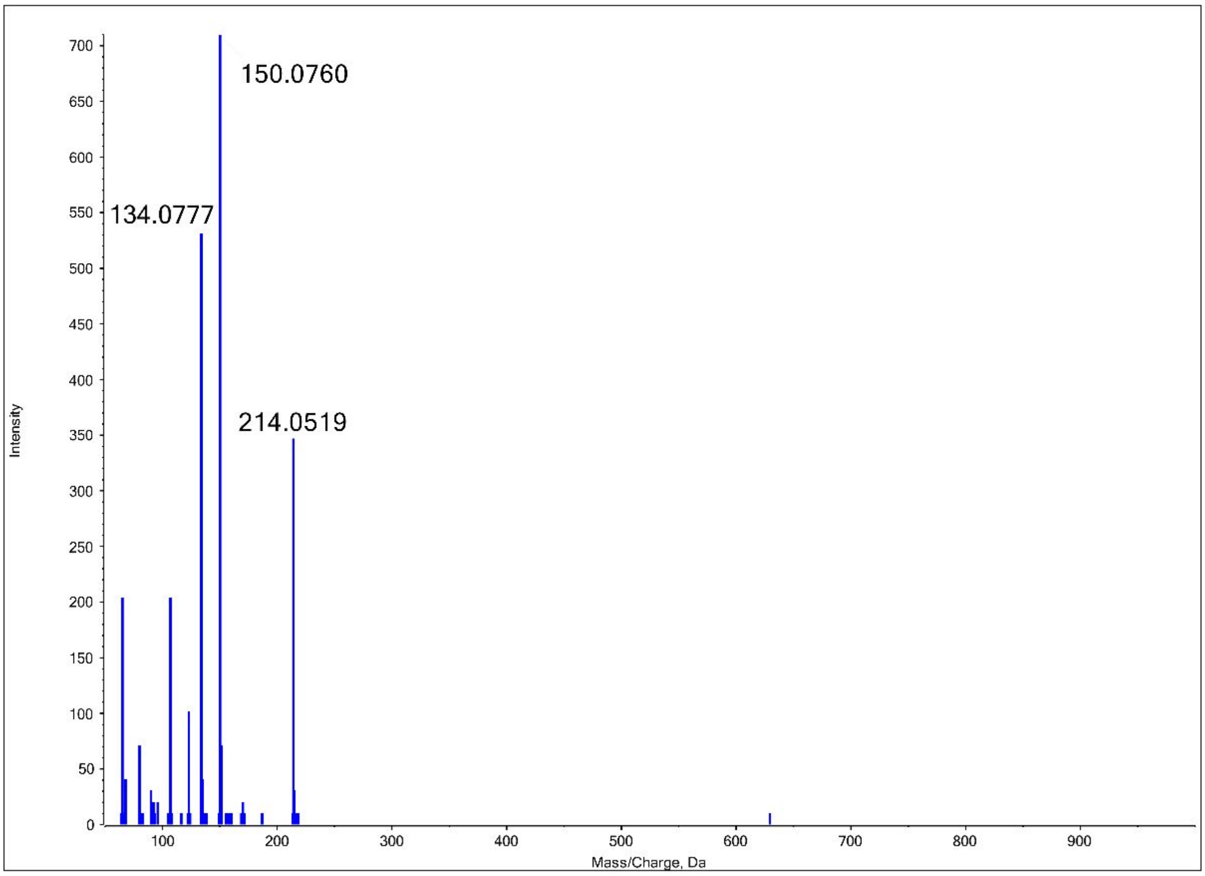


**Figure S8**.LC–MS/MS analysis of radiolysis product corresponding to the peak at 2.98 min on a HPLC chromatogram (see Figure 4) performed on a SCIEX TripleTOF 5600+ system equipped with an ESI source operated in negative ion mode. Product ion spectra were acquired in targeted MS/MS mode for predefined precursor ions at m/z = 214.0519. Nitrogen was used as the collision gas. TOF MS/MS spectra were recorded over the m/z 50–1000 range . MS/MS transitions of m/z 214.0519 (214.0519 → 150.0760 → 134.0777) correspond to the following transformations of the [ASCF3−HF−H]$^-$ pseudomolecular ion: [C6H2N5SF2]$^-$ → [C3H2N3SF2]$^-$ → [C3N2SF2]$^-$

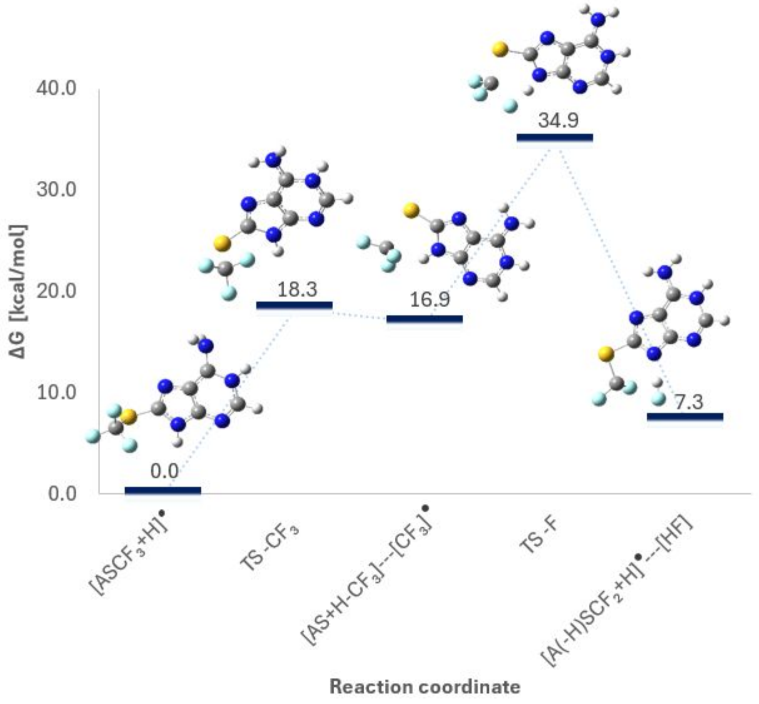


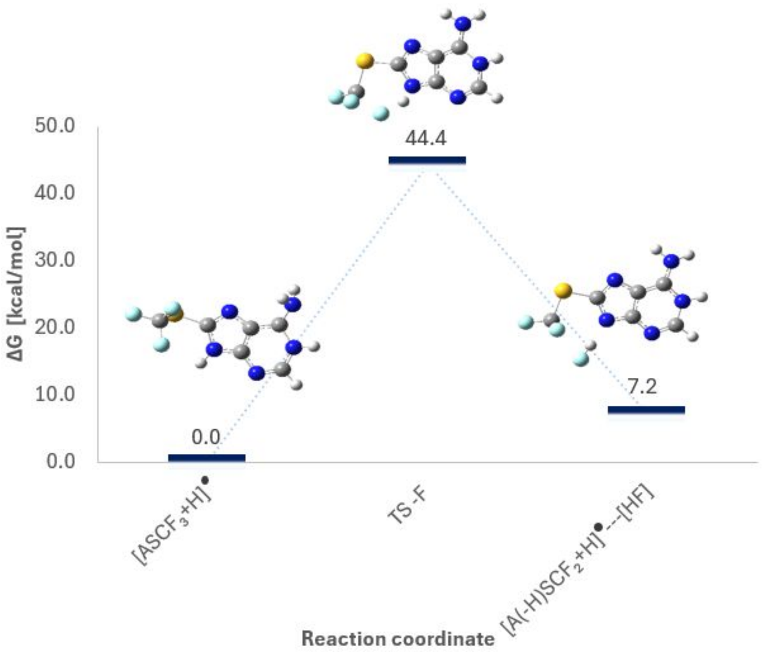


Figure S9. Free energy changes for the gas phase dissociative electron attachment (DEA) pathway of the protonated ASCF3 molecule, $[ASCF_3+H]^+$, calculated at the M06-2X/6-31++G(d,p) level of theory. The pathway is shown starting from the radical species formed upon  electron attachment) for clarity. The AEAG of $[ASCF_3+H]^+$ is 4.9 eV. The upper panel presents the stepwise elimination of HF, i.e. initial S-C bond dissociation followed by  C-F bond breakage, whereas the bottom panel shows the concerted HF elimination pathway.
